# Reactive strength index during single-limb vertical continuous jumps after anterior cruciate ligament reconstruction: cross-sectional study

**DOI:** 10.1186/s13102-022-00542-x

**Published:** 2022-08-02

**Authors:** Kenji Hirohata, Junya Aizawa, Takehiro Ohmi, Shunsuke Ohji, Sho Mitomo, Toshiyuki Ohara, Hideyuki Koga, Kazuyoshi Yagishita, Tetsuya Jinno, Atsushi Okawa

**Affiliations:** 1 Clinical Center for Sports Medicine and Sports Dentistry, Tokyo Medical and Dental University, 1-5-45 Yushima, Bunkyo-ku, Tokyo, 113-8519 Japan; 2 Department of Physical Therapy, Faculty of Health Science, Juntendo University, Tokyo, Japan; 3 Department of Rehabilitation Medicine, Graduate School of Medical and Dental Sciences, Tokyo Medical and Dental University, Tokyo, Japan; 4 Department of Joint Surgery and Sports Medicine, Graduate School of Medical and Dental Sciences, Tokyo Medical and Dental University, Tokyo, Japan; 5 Department of Orthopaedic Surgery, Dokkyo Medical University Saitama Medical Center, Saitama, Japan; 6 Department of Orthopaedic and Spinal Surgery, Graduate School of Medical and Dental Sciences, Tokyo Medical and Dental University, Tokyo, Japan

**Keywords:** ACL tear, Vertical jump performance, Asymmetry

## Abstract

**Background:**

The association of the reactive strength index (RSI) during single-limb vertical continuous jumps (SVCJs) with single-limb hop tests in athletes after anterior cruciate ligament reconstruction (ACLR) is unclear. Thus, this study aimed to confirm the measurement properties of the RSI during SVCJs in athletes with ACLR at the phase of determining the timing of their return to sport.

**Methods:**

RSI during SVCJs and single-limb hop (single, triple, and crossover) tests were measured for post-ACLR and healthy athletes. The limb symmetry index (LSI) was calculated using the measurements of each parameter. For each test, patients were divided into two subgroups according to their LSI score (≥ 90%, satisfactory; < 90%, unsatisfactory). Fisher’s exact test was used to examine the association of single-limb hop tests with RSI during the SVCJs.

**Results:**

A total of 21 post-ACLR and 17 healthy athletes completed all the tests. RSI during SVCJs was significantly lower on the involved limb than on the uninvolved limb in post-ACLR athletes (*P* < 0.001). The LSI of RSI during SVCJs of post-ACLR athletes was significantly lower than that of the healthy athletes (*P* < 0.01). Among the post-ACLR athletes, < 30% of those with LSIs > 90% in the single-limb hop tests had an LSI > 90% of the RSI during SVCJs.

**Conclusions:**

RSI during SVCJs of post-ACLR athletes was significantly lower on the involved limb than on the uninvolved limb, and the asymmetry was more remarkable in the SVCJs than in the single-limb hop tests.

## Background

Recently, a timing decision of return to sport is recommended depending on the performance as well as the time after an anterior cruciate ligament (ACL) injury and reconstruction [1–3]. Single-limb hop tests are among the primary tests and form part of the criteria for determining an athlete’s return to sport [4]. They include several tasks that involve hopping on a single limb to assess distance or speed [5, 6]. Of the traditional single-limb hop tests, three measure hopping distance: single hop for distance (SHD), triple hop for distance (THD), and crossover hop for distance (CHD) [6]. Single-limb hop tests, conducted periodically beginning three months after an ACL reconstruction (ACLR), can help monitor an athlete’s recovery [7]. Hop symmetry is assessed using the limb symmetry index (LSI) [8]. In athletes, an LSI above 90% for single-limb hop tests is one of the leading criteria for determining their return to sport after an ACLR [9]. After an ACLR, the LSI of the SHD has been reported to increase earlier than in other outcome measures, such as muscle strength [7]. These findings suggest that the LSIs of horizontal hop distance measured by single-limb hop tests may overestimate the involved limb’s function after an ACLR.

In addition to the tests that measure horizontal distances, such as single-hop tests, single-limb jump tests in the vertical direction were often used [10–12]. However, few reports evaluate jump performance in the vertical direction in post-ACLR athletes compared to those measuring the single-limb hop test [13]. Vertical jumps are frequently required in movements such as rebounding in basketball or blocking in volleyball [14, 15]. Effective landing from a vertical jump requires resisting external knee-flexion moment by activating muscles to absorb energy [16]. In post-ACLR athletes, sagittal-plane knee biomechanics of vertical direction jump-landing were related to knee muscles function [17]. Considering the above, any vertical jump test would be useful for post-ACL athletes. However, no generalized measurement protocol has been established to evaluate an athlete’s vertical jumping ability in the return to sport criteria after ACLR [18, 19].

Single-limb vertical continuous jumps (SVCJs) is one of the vertical jump tests used on post-ACLR athletes [10, 20]. After an ACLR, jump height during SVCJs is a parameter that can detect lower limb asymmetry in the single-limb jump performance of an athlete who is at the stage of potentially returning to sport [10]. Unlike the single-limb hop tests, SVCJs are not forward jumps; they involve continuous vertical jumps at the fastest pace possible [10, 20]. Myer et al. reported that the jump height during SVCJs of the involved limb of athletes was lower than that of the uninvolved limb over eight months after ACLR [10]. In the study by Myer et al., one of the analyzed parameters was the jump height during SVCJs [10]. In their research, the ground contact time was not analyzed, although it is typically analyzed in SVCJs in addition to jumping height when assessing jumping ability [21]. During tasks such as SVCJs, the duration of the contact time is related to the ground reaction force produced during the task [22]. Analyzing both the contact time and jump height in SVCJs will increase the likelihood of detecting asymmetry in the single-limb jumping ability. Contact time during SVCJs is one of the variables of stretch–shortening cycle capability [23–25]. The direct association is unclear, but stretch–shortening cycle capability may be associated with hyper joint motions during tasks, leading to soft tissue injury [26]. From the viewpoint of re-injury prevention, we consider it necessary to focus on variables including ground contact time.

In previous studies on healthy athletes, the reactive strength index (RSI), calculated using jump height and contact time, was used as a parameter for determining jumping ability [27, 28]. In addition to SVCJs, drop vertical jump (DVJ) and others are used in the movement to calculate the RSI. Studies of post-ACLR athletes used particular equipment movement [29] and DVJ [30]. However, no studies have investigated the RSI during SVCJs in athletes who have undergone an ACLR. In sports science, DVJ is a major movement. A box is required to measure DVJ, and RSI during exercise depends on the box height [31–33]. The box heights used in previous studies have varied, making it difficult to standardize protocols. In contrast, SVCJs does not require a box and have the advantage of a relatively standardized protocol. Moreover, no studies have compared the LSI of the RSI during SVCJs between post-ACLR and healthy athletes. In addition, the association of the LSI of the RSI during SVCJs with the LSI of the single-limb hop tests in athletes after ACLR is unclear. If the capability to detect functional asymmetry in the lower extremities of post-ACLR athletes can be confirmed, RSI during SVCJ could be a useful indicator in postoperative rehabilitation.

Thus, this study aimed to confirm the properties of the RSI during SVCJs and scores of single-limb hop tests to detect asymmetry in the single-limb jumping performance of post-ACLR athletes. Our hypotheses were as follows: (1) the RSI scores during SVCJs and single-limb hop tests of the ACL-reconstructed limb will be significantly lower than that of the contralateral limb. (2) The LSI scores of the RSI during SVCJs and single-limb hop tests will be significantly lower in post-ACLR athletes than in healthy athletes. (3) The LSI scores of the RSI during SVCJs and single-limb hop tests are related; however, among those with ≥ 90% score in the LSI of single-limb hop tests, some will have < 90% score in the LSI of the RSI during SVCJs.

## Methods

### Participants

In this study, we recruited athletes who had undergone a primary ACLR between July 2017 and March 2019 and healthy athletes aged 16–45 years at the time of measurement. The inclusion criteria for post-ACLR athletes were as follows: (1) participated in team sports that required multidirectional movements and jump landing (e.g., basketball, soccer, and lacrosse) with a modified Tegner activity scale score [34] of > 6 before the injury; (2) had undergone reconstruction at least 5 months ago; and (3) had undergone reconstruction using a bone-patellar tendon-bone graft, semitendinosus tendon, or semitendinosus tendon with additional gracilis tendon. Participants who had concomitant meniscal injuries were not excluded. The exclusion criteria for post-ACLR athletes were as follows: (1) had not participated in sports because of social reasons such as pregnancy and employment, (2) had an ACL injury to the contralateral knee or ACL re-injury to the reconstructed knee, (3) had a cartilage injury and/or other ligament injuries requiring surgery, and (4) had an injury that affected the physical function of the lower back or limb.

Healthy athletes were included in the study if they participated in a team sport that required multidirectional movements and jump-landing (e.g., basketball, soccer, and lacrosse) with a modified Tegner activity scale score of > 6. Healthy athletes with a previous ACL injury that required reconstruction and an injury that affected the physical function of the lower back or limb were excluded.

All power analyses were performed using G*power statistical software. For within-subject analyses, a priori power analysis using data from a pilot test (n = 10; involved limb RSI, 0.43 ± 0.14; uninvolved limb RSI, 0.52 ± 0.13; effect size, Cohen’s d = 0.665; alpha = 0.05; power = 0.80; two-tailed) revealed that at least 20 post-ACLR athletes were required to achieve an alpha of 0.05 and a power of 0.80. To compare the LSIs of the post-ACLR and healthy athletes, a power analysis using data from a previous study [35] with single-limb hop tests (SHD, THD, and CHD) revealed that at least 16 participants were required to achieve an alpha of 0.05 and a power of 0.80.

### Surgical technique

All surgeries were performed by orthopedic surgeons specializing in the knee joint. All ACLR athletes had a bone-patellar-bone or semitendinosus graft. An anatomical double-bundle reconstruction was performed using a semitendinosus tendon. If the semitendinosus graft alone was insufficient, the gracilis tendon was added.

### Postoperative rehabilitation protocols

All ACLR athletes received postoperative rehabilitation in accordance with the following protocol. However, squatting with a knee flexion beyond 90° was contraindicated in ACLR athletes who had undergone repair of the middle-posterior segment of the meniscus.

In the first weeks following reconstruction, ACLR athletes were encouraged to rest and limit activities of daily living to reduce knee swelling. A knee brace (Straighten Position Knee-Joint Immobilizer, ALCARE, Tokyo, Japan) and crutches were discontinued four weeks after reconstruction. Knee range of motion exercises from full extension to 120° of flexion started on the second day after reconstruction. Closed kinetic chain exercises such as weight shifting and squatting started between 1 and 2 weeks after reconstruction.

Jogging drills started at three months after reconstruction in athletes who had fulfilled the criterion of an LSI score of 65% of the knee extension strength at 60°/s. Once 80% of a subjective full-speed running ability was achieved, sport-specific exercises were initiated with detailed instructions. Participation in sport games was allowed at six months after reconstruction. The decision on return to sport was made by the treating surgeon based on the following criteria: no problematic symptoms in the knee joint, full range of motion, sufficient knee isokinetic flexion/extension strength at 60°/s (LSI > 85%), and sufficient single-limb hop ability measured by the single-limb SHD (LSI of the distance > 85%) after the athletes completed the specified athletic training.

### Demographic characteristics and sports activity level

On the same testing day, age, sex, height, and weight were recorded. The sports activity level was graded using the modified Tegner activity scale [34]. To score the modified Tegner activity scale, we checked the status of the healthy athletes and the pre-injury status of the ACLR athletes. The grades of futsal (lower division), lacrosse (lower division), and lacrosse (recreational level) were 8, 8, and 7, respectively.

### Single-limb hop tests

The following single-limb hop tests were performed: SHD, THD, and CHD [5]. Participants started each test with the lead toe behind a clearly marked starting line. No restrictions were placed on arm movement or gaze direction during the tests.

For a hop to be deemed successful, the landing had to be maintained. An unsuccessful hop was classified by any of the following: landing with an early touchdown of the contralateral limb, loss of balance, or an additional hop on landing. If the hop was unsuccessful, it was repeated. Participants were instructed to wear the footwear they would normally use during their training and sporting activities.

For the SHD, participants were instructed to stand on the limb to be tested, hop forward as far as possible, and land on the same limb [5, 6]. The distance from the starting line to the point of heel contact of the participant’s test limb upon completing the single hop was measured and recorded [8, 36]. For the THD, participants were instructed to stand on the limb to be tested, perform three consecutive maximal forward hops, and land on the same limb [5, 6]. For the CHD, participants were instructed to stand on the limb to be tested, perform three consecutive maximal forward hops while alternately crossing over a 15-cm marking strip on the floor [6]. When measuring the right limb, participants began the test by standing on the right limb on the right side of the line [36]. For the THD and CHD, the distance from the starting line to the point of heel contact of the participant’s test limb after completing the third hop was measured and recorded [36].

For each hop test, participants were initially given a verbal description of the test, and they were allowed to perform practice trials until they were confident to perform the test. Two trials were measured and recorded; the average of the two was used for analysis [5].

### SVCJs for measuring the RSI

For the SVCJs test, each participant was instructed to jump as high as possible with as little ground time as possible, land under control, recover his or her balance, and repeat the vertical jump 15 times. The SVCJs test was performed twice. From our pilot study, the within-session intraclass ability of the single-limb RSI measures using the described testing methods demonstrated high reliability. The intraclass correlation coefficient (1.1) values ranged from 0.83 to 0.96.

The Optojump Next system (Microgate, Bolzano, Italy) was used to measure the contact and flight time using a sampling rate of 1000 Hz. The system automatically calculates the jump height from the flight time using the following equation: jump height (m) = 9.81 × flight time^2^/8 [37, 38]. The unit of jump height was converted from meters to centimeters. The contact time and jump height were calculated from the average data of the middle 5 of the 15 jumps obtained during the test. The RSI was calculated based on the following equation: reactive strength index = jump height (cm) / contact time (s) [27, 39]. The average RSI of the two trials was used for analysis.

### Calculation of the limb symmetry index

In post-ACLR athletes, except for contact time, the LSI for each measurement variable was calculated by dividing the values of the involved limb by that of the uninvolved limb. For contact time, LSI was calculated by dividing the value of uninvolved limb by the value of involved limb. In healthy athletes, the LSI of each measurement variable was calculated by dividing the values of the poor-performing limb by that of the well-performing limb [40]. The LSI is expressed as a percentage value [41].

### Statistical analysis

All statistical analyses were performed using SPSS version 23.0 (SPSS, Chicago, IL, USA). Demographic statistics were generated for all variables. Independent t-test and chi-square test were used to evaluate differences between ACLR athletes and healthy athletes, with the significance level set at 5%. Paired t-tests were used to evaluate differences in measurement values of the limbs in both post-ACLR and healthy athletes. Independent t-tests were performed to assess differences in the LSI of each test between post-ACLR and healthy athletes, with the significance level set at 5%. Effect sizes were determined using Cohen’s d method, which defines 0.2, 0.5, and 0.8 as small, medium, and large, respectively [41].

For each test, patients were divided into two subgroups according to their LSI score (≥ 90%, satisfactory; < 90%, unsatisfactory) [9]. To examine the association of single-limb hop tests with the RSI during the SVCJs test, frequency distribution tables (2 × 2) were constructed and Fisher’s exact test was used to test for significant associations. A *P* value of < 0.05 was considered statistically significant for the Fisher’s exact test.

## Results

### Participants

In this study, 21 post-ACLR and 17 healthy athletes completed the single-limb hop and SVCJs tests. The demographics of the participants are shown in Table 1. For the post-ACLR athletes, the mean duration after reconstruction was 11.6 ± 6.9 (range, 6.7–31.2) months.

**Table 1:** Participants’ demographics

Variables	Participants		*P* value
	Post-ACLR athletes	Healthy athletes	
	n = 21	n = 17	
Age at measurement, year	20.9 ± 3.6 (16–30)^a^	19.8 ± 2.8 (16–27)^a^	0.369
Sex, n (%)			
Male	5 (23.8)	5 (29.4)	0.697
Female	16 (76.1)	12 (70.6)	
Modified Tegner activity score	7.3 ± 0.2 (6–10)^a^	7.8 ± 0.2 (7–10)^a^	0.168
Graft type, n (%)			
Semitendinosus tendon graft	16 (76.1)	–	
Semitendinosus with additional gracilis tendon	2 (9.5)	–	
Bone-patellar tendon-bone graft	3 (14.3)	–	
Meniscus injury, n (%)			
Yes	17 (80.9)	–	
No	4 (19.0)	–	
Meniscus treatment, n (%)			
Lateral meniscus	13 (61.9)	–	
Medial meniscus	8 (38.1)	–	
None	3 (14.3)	–	
Time from surgery to measurement, month	11.6 ± 6.9 (6.7–31.2)^a^	–	

ACLR, anterior cruciate ligament reconstruction

^a^Reported as mean ± standard deviation (range)

### Side-to-side differences within individuals by group

The results of the within-subject analysis are shown in Table 2. In the post-ACLR athletes, the distance of the single-limb hop tests and RSI during the SVCJs test of the involved limb was significantly lower than that of the uninvolved limb. Compared with the single-limb hop tests, the RSI during the SVCJs test showed a higher effect size. In healthy athletes, the distance of the single-limb hop tests and RSI during the SVCJs test was significantly lower in the poor-performing limb than in the well-performing limb.

**Table 2:** Side-to-side differences for each test

Variables	Post-ACLR athletes (n = 21)		*P* value	Effect size(d)	Healthy athletes (n = 17)		*P* value	Effect size (d)
	Involved limb mean ± SD (range)	Uninvolved limb mean ± SD (range)			Poor-performing limb mean ± SD (range)	Well-performing limb mean ± SD (range)		
SHD (cm)	135.1 ± 26.8 (99.5–191.5)	142.1 ± 26.2 (97.5–195.0)	0.006	0.26	136.6 ± 24.2 (101.0–188.5)	143.5 ± 26.6 (103–197.5)	< 0.001	0.27
THD (cm)	407.2 ± 69.6 (286.5–557.5)	429.1 ± 72.3 (315.0–633.0)	0.001	0.31	450.7 ± 67.3 (365.0–607.0)	471.0 ± 68.6 (389.5–614.5)	< 0.001	0.30
CHD (cm)	354.5 ± 64.3 (257.5–493.0)	379.1 ± 73.7 (261.0–517.5)	< 0.001	0.36	401.5 ± 68.7 (293.5–555.0)	419.1 ± 71.9 (304.0–556.5)	< 0.001	0.25
CT during SVCJs (sec)	0.26 ± 0.03 (0.21–0.33)	0.25 ± 0.03 (0.21–0.30)	< 0.001	0.33	0.24 ± 0.02 (0.19–0.27)	0.23 ± 0.02 (0.19–0.26)	< 0.001	0.50
JH during SVCJs (cm)	10.1 ± 2.4 (6.9–17.7)	11.9 ± 3.1 (7.8–20.7)	< 0.001	0.65	12.5 ± 3.4 (7.6–22.2)	13.6 ± 3.4 (8.6–22.4)	< 0.001	0.32
RSI during SVCJs (cm/s)	38.8 ± 9.5 (25.4–65.0)	48.4 ± 11.4 (26.6–73.0)	< 0.001	0.92	52.3 ± 13.7 (27.9–88.2)	57.7 ± 14.0 (33.4–90.5)	< 0.001	0.39

ACLR, anterior cruciate ligament reconstruction; SD, standard deviation; SHD, single hop for distance; THD, triple hop for distance; CHD, crossover hop for distance; CT, contact time; SVCJs, single-limb vertical continuous jumps; JH, jump height; RSI, reactive strength index

### Comparison of the LSIs of post-ACLR and healthy athletes

The LSIs of each test are shown in Table 3. Only the LSIs calculated from the contact time and RSI during the SVCJs test were significantly lower in the post-ACLR athletes than in the healthy athletes.

**Table 3:** Limb symmetry index for each test

Variables	Participants		*P* value	Effect size (d)
	Post-ACLR athletes (n = 21) mean ± SD (range)	Healthy subjects (n = 17) mean ± SD (range)		
SHD (%)	95.0 ± 6.7 (85.3–117.8)	95.4 ± 2.6 (91.1–99.7)	0.900	0.08
THD (%)	94.9 ± 5.5 (83.7–108.3)	95.7 ± 3.4 (87.9–99.3)	0.631	0.17
CHD (%)	93.9 ± 5.8 (82.1–103.7)	95.9 ± 2.6 (91.3–99.9)	0.184	0.43
CT during SVCJs (%)	94.3 ± 4.9 (86.6–103.9)	97.0 ± 2.4 (90.0–99.9)	0.034	0.68
JH during SVCJs (%)	86.0 ± 12.8 (66.9–120.8)	91.6 ± 5.6 (79.6–100.0)	0.106	0.55
RSI during SVCJs (%)	81.4 ± 14.8 (58.1–125.5)	90.6 ± 5.9 (78.8–98.9)	0.015	0.79

ACLR, anterior cruciate ligament reconstruction; SD, standard deviation; SHD, single hop for distance; THD, triple hop for distance; CHD, crossover hop for distance; CT, contact time; SVCJs, single-limb vertical continuous jumps; JH, jump height; RSI, reactive strength index

### Frequency distribution table analysis of post-ACLR athletes

In this study, 75%–80% of the post-ACLR athletes had been judged “satisfactory” based on the LSIs from the single-limb hop tests. By contrast, only 25% (5/21) had been judged “satisfactory” based on the LSI from the RSI during the SVCJs test (Table 4). Recovery of satisfactory RSI during the SVCJs test was associated with the symmetry of the single-limb hop tests. Of the patients who had an LSI ≥ 90% from the RSI during the SVCJs test, 100% (5/5), 80% (4/5), and 60% (3/5) also had an LSI ≥ 90% for SHD, THD, and CHD, respectively.

**Table 4:** Association of the single-limb hop tests with the RSI during the SVCJs test in post-ACLR athletes

	LSI	Single-limb hop tests											
		SHD				THD				CHD			
		≥ 90%	< 90%	Sum	*P* value	≥ 90%	< 90%	Sum	*P* value	≥ 90%	< 90%	Sum	*P* value
RSI during SVCJs	≥ 90%	3	0	3	< 0.01	3	0	3	< 0.01	3	0	3	< 0.01
	< 90%	15	3	18		15	3	18		13	5	18	
	Sum	18	3	21		18	3	21		16	5	21	

RSI, reactive strength index; SVCJs, single-limb vertical continuous jumps; ACLR, anterior cruciate ligament reconstruction; SHD, single hop for distance; THD, triple hop for distance; CHD, crossover hop for distance

Recovery in the single-limb hop tests was associated with the RSI during the SVCJs test. However, only 29.4% (5/17), 23.5% (4/17), and 18.8% (3/16) of the athletes who had satisfactory recovery for SHD, THD, and CHD, respectively, had satisfactory recovery for RSI during the SVCJs test.

## Discussion

In this study, the RSI during SVCJs and the scores of single-limb hop tests were significantly lower for the involved limb than for the uninvolved limb in post-ACLR athletes, and they showed the largest effect size for the difference in the RSI during SVCJs. No significant difference was found in the LSIs of single-limb hop tests between post-ACLR and healthy athletes. By contrast, the RSI during SVCJs was significantly lower in post-ACLR athletes than in healthy athletes, and the effect size was large. Among post-ACLR athletes, less than 30% of those with LSIs above 90% in the single-limb hop tests had LSIs above 90% in the RSI during SVCJs. These results support our hypotheses, except for the difference in the LSIs between the groups in the single-limb hop tests.

Gokeler et al. reported a significant difference in SHD measurements between the involved and uninvolved limbs of post-ACLR athletes [42]. Herrington et al. reported similar findings for the THD and CHD measurements with a moderate effect size [43]. Our results generally support these reports, but the effect sizes were relatively small. These previous studies have reported duration of 6.7–7.8 months since reconstruction in post-ACLR athletes. Our study contradicts these findings and reports a mean duration of 11.6 (6.7–31.2) months. The LSIs of the single-limb hop tests that measure distance increased as duration increased after reconstruction [44]. The longer duration after reconstruction in our study may have influenced the difference of effect sizes observed in the results between the present study and previous studies.

In this study, the RSI during SVCJs was significantly lower on the involved limb than on the uninvolved limb of post-ACLR athletes and had a large effect size. Their corresponding LSIs were significantly lower than that of healthy athletes, and the effect size was large. Myer et al. reported that the mean LSI of the jump height of post-ACLR athletes was 89% [10]. Similarly, the mean jump height and RSI during SVCJs in the present study was 86% and 81%, respectively. The study findings show that greater lower limb asymmetry can be detected by calculating the RSI with the jump height and contact time. In many previous studies that have assessed jump with landing and leaping for healthy athletes, the contact time and RSI have been used as parameters of jump performance [45, 46]. The RSI for continuous vertical jumps in post-ACLR athletes is not known. Therefore our study results provide new insights into the RSI during SVCJs in post-ACLR athletes who are determining the appropriate timing to return to sport.

Less than 30% of the post-ACLR athletes with an LSI of 90% or above in the single-limb hop tests had an LSI over 90% in the RSI during SVCJs. This study showed that the symmetry of single-limb forward hop performance was restored in post-ACLR athletes more than six months after surgery. Still, asymmetry remained in their reactive continuous vertical jump performance. According to a study that analyzed the LSIs of the jump height during SVCJs, SHD, and THD in post-ACLR athletes 54 weeks after reconstruction, the LSI of the jump height during SVCJs was the lowest, which is similar to our study findings [47]. The present results support our hypothesis. For post-ACLR athletes at the phase of determining the timing of their return to sport, the RSI during SVCJs was shown to detect lower limb asymmetry more easily than the single-limb hop tests.

In our study, a side-to-side difference was found in the single-limb hop tests and RSI during SVCJs in both post-ACLR and healthy athletes. In healthy athletes, LSI for each test ranged from 90.6% to 95.9%. Several studies have reported no or slight side-to-side differences in single-limb hop tests in healthy athletes, with LSIs ranging from 94.5 to 100% [48, 49]. A difference was found in the methods of LSI calculations, and various definitions were used to categorize the lower limbs between the studies. Some studies mostly analyzed the differences and ratios between the dominant and non-dominant limbs separately in healthy athletes [48, 50, 51]. Our study divided the lower limbs of healthy athletes into poor-performing and well-performing limbs. Therefore, we observed a greater difference when comparing the dominant and non-dominant limbs. In our study, the LSI was calculated using results of the poor-performing limb as the numerator and results of the well-performing limb as the denominator, which was effective in identifying the asymmetry in the limbs.

A decrease in leg stiffness associated with RSI score is one of the risks for soft tissue injury occurrence [26]. Recently, an association between lower RSI score and the occurrence of primary ACL injury was reported [52]. Although the measurement task was different from the current study, prolonged contact time during double-limb DVJ has been shown to be associated with future re-injury by King et al. [53]. Although additional research is needed, RSI including contact time may be useful in screening for re-injury risk. Furthermore, RSI is associated with measures of sports performance parameters [54, 55]. And RSI is a variable frequently used to determine the effectiveness of plyometric training [46, 56]. In general, plyometrics is defined as an exercise with a contact time of around 250 ms [57]. In this study, the average contact time averaged about 250 ms, which means that the SVCJs is a stretch–shortening cycle activity. RSI during SVCJs may provide insight into the recovery of plyometrics performance of athletes after ACLR, which cannot be obtained by horizontal distance measurements.

A recent systematic review identified the need for modification of return to sport criteria for adequate decision making regarding the timing of return to sport in post-ACLR athletes [13, 58]. Even if a test is able to accurately capture an athlete’s recovery status, generalization is difficult if it is not feasible in a clinical setting. Recent advances in technology have made it possible for relatively inexpensive devices such as mat switches [59] and smartphone applications [60] to accurately measure flight time and RSI for vertical jumps involving landing and leaping. The RSI during SVCJs employed in this study has the following advantages as a functional assessment of post-ACLR athletes. First, similar to the single-limb hop tests, this is a single-limb task; therefore, the asymmetry of the lower limb function can be confirmed. Second, SVCJs can be performed in a relatively space-saving manner. The results of this study indicate that the RSI during SVCJs may serve as a new indicator for detecting lower extremity asymmetry in post-ACLR athletes at the phase of determining the timing of their return to sport.

This study has several limitations. First, although sex influences the effect of lower body explosiveness on jump landings as assessed by the RSI [61], it was not included in the analysis. Approximately 70% of the study participants were female. Second, the surgical technique for inclusion was not limited. Third, because the study included post-ACLR athletes who were within 6 months to 2 years after surgery, the return to sport status varied, but it was not analyzed separately for timing or return to sport status. Fourth, we only used the Optojump™ system for the RSI during SVCJs measurements. In RSI measurements, fixed bias may occur due to differences in measurement equipment [28]. Therefore, careful attention is needed when comparing the data from this study with studies that have used different measuring instruments. Fifth, the heterogeneous time since reconstruction between the current and previous studies [42, 43]. Differences in time from surgery to measurement may affect the results. Sixth, no post-hoc adjustments were made for multiple testing. However, the RSI during SVCJs focused on in this study shows a sufficiently large effect size for both within-individual and between-group comparisons. Therefore, we think that the data of this study are clinically meaningful. Finally, data from healthy athletes are often analyzed separately for dominant and non-dominant limbs [48, 50, 51]. However, we segregated the data in this study into poorly-performing and well-performing limbs [40, 62]. RSI is a variable that can be altered by limb dominance [63]. Additional analysis is needed to examine the impact of limb dominance.

## Conclusion

The RSI during SVCJs on the involved side 6 months after reconstruction was significantly lower than that on the uninvolved side of post-ACLR athletes, and the asymmetry was more remarkable in the SVCJs than in the single-limb hop tests. Among the post-ACLR athletes who recovered the asymmetry in the single-limb hop tests, most had residual asymmetry in the RSI during SVCJs. These findings will assist in selecting an appropriate jumping performance test, which is an important component of the return to sport criteria for determining the timing of an athlete’s return to sport.

## Data Availability

The datasets used and/or analysed during the current study are available from the corresponding author on reasonable request.

## Abbreviations

RSI: Reactive strength index
SVCJs: Single-limb vertical continuous jumps
ACL: Anterior cruciate ligament
ACLR: Anterior cruciate ligament reconstruction
LSI: Limb symmetry index
SHD: Single hop for distance
THD: Triple hop for distance
CHD: Crossover hop for distance
LSI: Limb symmetry index
DVJ: Drop vertical jump
